# Hybridization capture-based next generation sequencing reliably detects *FLT3* mutations and classifies *FLT3*-internal tandem duplication allelic ratio in acute myeloid leukemia: a comparative study to standard fragment analysis

**DOI:** 10.1038/s41379-019-0359-9

**Published:** 2019-08-30

**Authors:** Rong He, Daniel J. Devine, Zheng Jin Tu, Ming Mai, Dong Chen, Phuong L. Nguyen, Jennifer L. Oliveira, James D. Hoyer, Kaaren K. Reichard, Paul L. Ollila, Aref Al-Kali, Ayalew Tefferi, Kebede H. Begna, Mrinal M. Patnaik, Hassan Alkhateeb, David S. Viswanatha

**Affiliations:** 1 Division of Hematopathology, Mayo Clinic College of Medicine, Rochester, MN USA; 2 Biomedical statistics and informatics, Mayo Clinic College of Medicine, Rochester, MN USA; 3 Division of Hematology, Mayo Clinic College of Medicine, Rochester, MN USA; 4 Present Address: Department of Laboratory Medicine, Cleveland Clinic, Cleveland, OH USA

**Keywords:** Acute myeloid leukaemia, Prognostic markers

## Abstract

*FLT3*-internal tandem duplication occurs in 20–30% of acute myeloid leukemia and confers an adverse prognosis with its allelic ratio being a key risk stratifier. The US Food and Drug Administration recently approved FLT3 inhibitors midostaurin and gilteritinib in *FLT3* mutation-positive acute myeloid leukemia. Historically, *FLT3* was tested by fragment analysis, which has become the standard method endorsed by international guidelines. However, next generation sequencing is increasingly used at acute myeloid leukemia diagnosis given its ability to simultaneously evaluate multiple clinically informative markers. As *FLT3*-internal tandem duplication detection was known to be challenging by next generation sequencing and the results carry profound prognostic and therapeutic implications, it is important to thoroughly examine its performance in *FLT3-*internal tandem duplication detection and allelic ratio classification. In a comparative study with fragment analysis, we retrospectively reviewed our experience using a custom-designed, hybridization capture-based, targeted next generation sequencing panel. Among 7902 cases, *FLT3*-internal tandem duplication was detected in 335 with variable sizes (3–231 bp) and insertion sites. Fragment analysis was also performed in 402 cases, demonstrating 100% concordance in *FLT3*-internal tandem duplication detection. In 136 dual-tested, positive cases, 128/136 (94%) exhibited concordant high/low allelic ratio classifications. The remaining 6% showed borderline low allelic ratio by next generation sequencing. The two methods were concordant in *FLT3*-tyrosine kinase domain mutation detection at the hotspot D835/I836 targeted by fragment analysis. Furthermore, seven mutations which may benefit from FLT3 inhibitor therapy were detected by next generation sequencing, in regions not covered by fragment analysis. Our study demonstrates that using a hybridization capture-based chemistry and optimized bioinformatics pipeline, next generation sequencing can reliably detect *FLT3*-internal tandem duplication and classify its allelic ratio for acute myeloid leukemia risk stratification. Next generation sequencing also exhibits superior comprehensiveness in *FLT3* mutation detection and may further improve personalized, targeted therapy in acute myeloid leukemia.

## Introduction

FMS-like tyrosine kinase 3 (FLT3) is a class III receptor tyrosine kinase playing important roles in hematopoietic stem cell survival, proliferation, and differentiation. It activates downstream signaling pathways including RAS, AKT1, ERK, and mTOR [1–4]. *FLT3*-internal tandem duplication occurs in ~20–30% of acute myeloid leukemia resulting in constitutive activation and abnormal cellular proliferation [1, 5]. *FLT3*-internal tandem duplication is associated with adverse prognosis in acute myeloid leukemia, particularly in patients with normal or intermediate risk karyotype [6–9]. The allelic ratio of *FLT3*-internal tandem duplication to wild-type *FLT3* has been shown to correlate with clinical outcome, and a cutoff value of 0.5 was adopted by both the European Leukemia Net and National Comprehensive Cancer Network consensus guidelines to classify *FLT3-*internal tandem duplication as high and low allelic ratio categories and further risk stratify *FLT3-*internal tandem duplication-positive acute myeloid leukemia patients in the context of *NPM1* and cytogenetic findings [10, 11]. *FLT3*-tyrosine kinase domain mutations occur in ~7% of acute myeloid leukemia. Their prognostic impact is less well-defined, although may confer acquired resistance to type II FLT3 inhibitors [12–14]. Recent years have witnessed rapid preclinical development and clinical advancement in targeted therapy. Through affinity to active or inactive receptor conformation, type I and type II FLT3 inhibitors inhibit *FLT3*-internal tandem duplication/tyrosine kinase domain mutation and *FLT3*-internal tandem duplication, respectively [13]. The US Food and Drug Administration recently approved type I inhibitors midostaurin and gilteritinib for treatment of adult acute myeloid leukemia patients harboring an *FLT3* mutation [15–17].

As *FLT*3-internal tandem duplication is a critical risk marker that directly impact prognostication and clinical management in acute myeloid leukemia [10, 11], and there is established clinical benefit of FLT3 inhibitor therapy in patients harboring an *FLT3* mutation [15–17], *FLT3* testing has become routine in the work-up of newly diagnosed acute myeloid leukemia, and likely will soon become standard for relapsed/refractory acute myeloid leukemia patients. Historically, this has been done using electrophoretic fragment sizing analysis following polymerase chain reaction (PCR) with primers flanking *FLT3* exons 14 and 15, and the *FLT3*-internal tandem duplication allelic ratio was obtained from the fluorescence intensity ratio of the *FLT3*-internal tandem duplication versus wild-type peaks [18, 19]. Fragment analysis has gradually become the standard method for *FLT3* testing over the years, and was endorsed by the European Leukemia Net and National Comprehensive Cancer Network consensus guidelines for *FLT3*-internal tandem duplication allelic ratio evaluation in acute myeloid leukemia risk stratification. The US Food and Drug Administration also approved a companion diagnostic test for midostaurin and gilteritinib using fragment analysis. At the same time, with the advent of next generation sequencing, clinical laboratories have increasingly adopted next generation sequencing as a more comprehensive acute myeloid leukemia genetic testing method, given its ability to simultaneously evaluate multiple clinically informative markers, such as *NPM1, CEBPA*, *KIT*, *TP53*, *AXSL1*, *RUNX1*, *IDH1*, and *IDH2* [20]. However, *FLT3*-internal tandem duplication is a difficult-to-detect entity by next generation sequencing given its heterogeneity in size (3–400 bp), insertion site and perfect/near-perfect duplication of wild-type sequence, posing challenges for informatics processing and analysis [21–23]. Although many next generation sequencing panels include *FLT3*, their performance in detecting *FLT3-*internal tandem duplication and their accuracy of assessing *FLT3-*internal tandem duplication allelic ratio for acute myeloid leukemia risk stratification has not been thoroughly examined in comparison with the standard fragment analysis assay. Given the prognostic and therapeutic clinical impact of *FLT3* mutation detection, we herein report our experience of *FLT3* testing using a custom-designed, hybridization capture-based, targeted next generation sequencing panel, and its comparison with the standard fragment analysis test.

## Materials and methods

### Case selection

Following Mayo Clinic Institutional Review Board approval, we retrospectively reviewed the results of a targeted 35-gene OncoHeme next generation sequencing panel performed at our clinical Molecular Hematopathology Laboratory from 7/2015 to 9/2018. Concurrent single gene *FLT3* test results by fragment analysis were collected in these samples. Fragment analysis test was also retrospectively performed on 95 available samples submitted for next generation sequencing testing. Diagnoses were collected by clinical chart review for Mayo Clinic patients, and the indication for next generation sequencing testing was retrieved for cases submitted for testing from outside institutions.

### OncoHeme next generation sequencing panel test

The OncoHeme next generation sequencing panel interrogates 35 genes recurrently mutated in myeloid neoplasms, including *ASXL1, BCOR, BRAF, CALR, CBL, CEBPA, CSF3R, DNMT3A, ETV6, EZH2, FLT3, GATA1, GATA2, IDH1, IDH2, JAK2, KIT, KRAS, MPL, MYD88, NOTCH1, NPM1, NRAS, PHF6, PTPN11, RUNX1, SETBP1, SF3B1, SRSF2*, *TERT, TET2, TP53, U2AF1, WT1*, and *ZRSR2*. DNA was extracted from bone marrow/peripheral blood samples from patients with known/suspected hematologic neoplasms. Next generation sequencing was performed using 200 ng sheared DNA with a custom hybridization-capture reagent (SureSelect^XT^, Agilent, Santa Clara, CA) and sequenced on the MiSeq or HiSeq platform (Illumina, San Diego, CA). The read length was 151 and 101 bp on the Miseq and Hiseq platforms, respectively. Sequencing data were processed through a custom bioinformatics pipeline Mayo Next Generation Sequencing Workbench (Workbench), using CLC Bio Genomics Server v6.0 (Qiagen, Redwood City, CA) for alignment and variant calling. The aligned Binary Alignment Map files were further processed through an in-house developed breakpointSearch tool for large insertion/deletion detection (please contact Dr Zheng Jin Tu at tuz@ccf.org for inquiries regarding this tool). It searches the Binary Alignment Map files for reads with soft-clipped bases and then do de novo assembly via the CAP3 program [24]. The assembled contigs were then aligned to the hg19 reference genome via the NCBI-BLAST program (Supplementary Fig. 1). The detected breakpoints were reported in Workbench. Binary Alignment Map files of all variant calls and breakpoints reported in Workbench were reviewed in the genome browser Alamut® Visual (Interactive Biosoftware, Rouen, France) for confirmation. *FLT3* (NM_004119.2) regions tested encompass exons 14–20 and intron 14. Exons 14 through 15 were also manually reviewed in Alamut® Visual in all cases given the clinical significance of *FLT3*-internal tandem duplication. This review serves to confirm the presence/absence of *FLT3*-internal tandem duplication reported by the breakpointSearch tool, to confirm the *FLT3*-internal tandem duplication variant calls made by the CLC Bio (internal tandem duplication <30 bp), and to make the final *FLT3*-internal tandem duplication variant call in large internal tandem duplications (≥30 bp). *FLT3*-internal tandem duplication variant allele fraction was estimated from the internal tandem duplication reads divided by the depth of total reads over the exon 14–15 region. *FLT3*-internal tandem duplication allelic ratio by next generation sequencing was calculated from the number of sequencing reads showing internal tandem duplication versus the wild-type sequence. It can also be calculated from the variant allele fraction using equation (variant allele fraction × 100)/(100 − variant allele fraction × 100), i.e., an FLT3-internal tandem duplication variant allele fraction of 33% equates to 33% FLT3-internal tandem duplication and 67% wild type, and therefore an allelic ratio of 0.5 (33/67).

### Single gene *FLT3* test by capillary electrophoretic fragment analysis

The fragment analysis test assessing *FLT3*-internal tandem duplication and the D835/I836 tyrosine kinase domain hotspot mutations was performed using PCR and capillary gel electrophoresis fragment sizing as previously described [18, 19, 25], except for D835/I836 testing, the following primers were used: F, 5′-GCCGTRAGAAAGATTGCACTCCAGGATAAT-3′; R, 5′-GCCGTATAAAAATAAGTAGGAA-3′. For *FLT3*-internal tandem duplication testing, primers flanking exons 14 and 15 were used for PCR followed by fragment analysis. Allelic ratio by fragment analysis was calculated from the fluorescent intensity ratio of the *FLT3*-internal tandem duplication versus wild-type peaks. For *FLT3*-tyrosine kinase domain D835/I836 testing, exon 20 PCR product was digested with EcoRV (New England Biolabs, Ipswich, MA), which recognizes a palindromic restriction site coincident with the D835/I836 coding sequence, followed by fragment analysis.

### Statistical analysis

Metric variable correlation between allelic ratios obtained by fragment analysis versus next generation sequencing and between the degree of allelic ratio difference and internal tandem duplication size was evaluated using the Pearson correlation test. Result was considered statistically significant at a level of *p* < 0.05.

## Results

### Clinical and molecular characteristics of *FLT3*-internal tandem duplication-positive cases identified by next generation sequencing

Among 7902 next generation sequencing cases tested between 7/2015 and 9/2018, *FLT3*-internal tandem duplication was detected in 335 cases. One hundred and fourteen positive cases were from Mayo Clinic patients including acute myeloid leukemia (*n* = 104), myelodysplastic/myeloproliferative neoplasm (*n* = 9), and early T-cell precursor lymphoblastic lymphoma (*n* = 1). The remaining 221 cases were received specimens from outside institutions and the indications for next generation sequencing testing included acute myeloid leukemia (*n* = 211), myeloid neoplasm (*n* = 1), myelodysplastic/myeloproliferative neoplasm (*n* = 2), myeloproliferative neoplasm (*n* = 1), cytopenia (*n* = 4), and leukocytosis (*n* = 2).

**Fig. 1:**
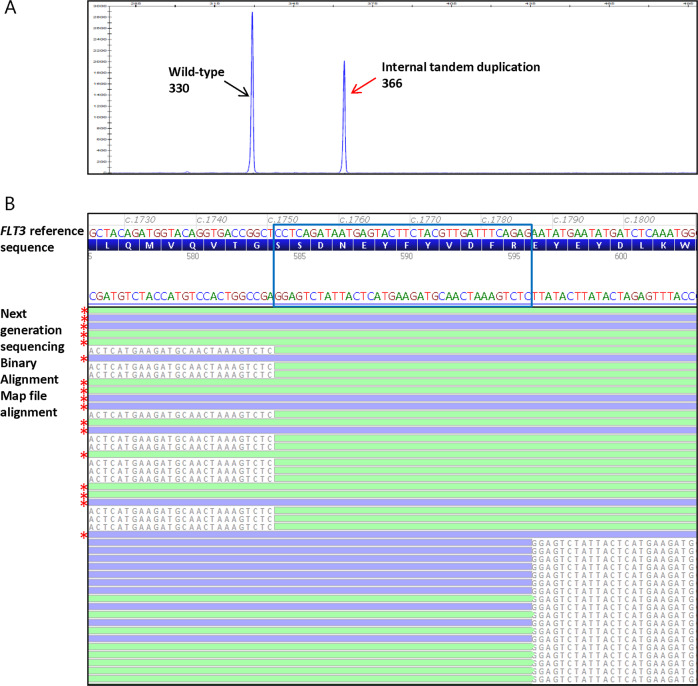
A representative acute myeloid leukemia case harboring an *FLT3*-internal tandem duplication tested by fragment analysis and next generation sequencing. **a** *FLT3* mutational analysis by fragment analysis test showing the presence of an *FLT3*-internal tandem duplication; *x* axis represent the PCR product size in base pair (bp) and *y* axis represent the fluorescence intensity. The wild-type peak at 330 bp (black arrow) and *FLT3*-internal tandem duplication peak at 366 bp (red arrow) were indicated. The *FLT3*-internal tandem duplication/wild-type allelic ratio was 0.7 as calculated from the internal tandem duplication and wild-type peak fluorescence intensity ratio. **b** Next generation sequencing read alignments displayed in Alamut® Visual demonstrating the presence of an *FLT3*-internal tandem duplication (c.1751_1786dup; p.R595_E596ins12) with a variant allele fraction (*FLT3*-internal tandem duplication/total) of 40% and a corresponding allelic ratio (*FLT3*-internal tandem duplication/wild type) of 0.7. *FLT3*-internal tandem duplication reads showed only partial alignment with the reference sequence and 5′ and 3′ soft-clipped bases (colored gray) flanking the duplicated wild-type sequence of the *FLT3*-internal tandem duplication (blue box). The 3′ soft-clipped bases mark the *FLT3*-internal tandem duplication insertion site and the *FLT3*-internal tandem duplication sequences. Red stars mark the wild-type reads with complete alignment with the reference sequence. Green, forward reads; blue, reverse reads. Note that the *FLT3* coding reference sequence is in reverse complementary orientation to the next generation sequencing Binary Alignment Map alignment sequence

*FLT3*-internal tandem duplications were clearly identifiable in the next generation sequencing Binary Alignment Map files as an insertional event (<30 bp) or an excess of partially aligned reads with soft-clipped bases flanking the duplicated wild-type sequence of the *FLT3*-internal tandem duplication (≥30 bp) adjacent to the insertion site (Fig. 1). To safeguard detection of large insertion/deletions, we developed the breakpointSearch tool to enrich reads containing soft-clipped bases followed by *de novo* assembly for breakpoints detection. Manual review of the *FLT3*-internal tandem duplication region in all cases showed that this tool successfully detected all *FLT3*-internal tandem duplication events in our study cohort. Overall, there were 286 distinct types of *FLT3*-internal tandem duplication, ranging in size from 3 up to 231 bp, with 268 (94%), 15 (5%), and 3 (1%) of 286 falling in the range of <100, 100–200, and >200 bp, respectively. In addition, 243 (85%), 8 (3%), and 35 (12%) internal tandem duplications showed insertion sites at exon 14, exon 15, and intron 14, respectively. Similar to previous reports [8, 18, 26], we observed simple internal tandem duplication, as well as insertion of random extra nucleotides in the 286 types of *FLT3*-internal tandem duplications: 154 (54%) demonstrated simple tandem duplications with a size range of 3–213 bp; and the other 132 had insertion of extra nucleotides of unknown origin, including 126 (44%) with insertions of 1–26 nucleotides in between the duplicated sequences (2–229 bp) and 6 (2%) with sole insertions of 18–36 nucleotides. Of the 335 cases harboring one or more *FLT3*-internal tandem duplications, 269 (80%), 56 (17%), 8 (2%), and 2 (1%) cases showed 1, 2, 3, and 4 internal tandem duplications, respectively (data not shown).

### Comparison of *FLT3*-internal tandem duplication detection by next generation sequencing and fragment analysis

Fragment analysis results were available in 402 of the 7902 next generation sequencing cases. In these dual-tested cases, next generation sequencing demonstrated 100% concordance with fragment analysis in *FLT3*-internal tandem duplication detection, with all 136 next generation sequencing-positive (size range 3–231 bp) and 266 next generation sequencing-negative cases consistently positive and negative by fragment analysis, respectively. All 136 *FLT3*-internal tandem duplication-positive cases demonstrated a confirmed diagnosis (internal cases) or clear test indication (outside cases) of acute myeloid leukemia.

### Comparison of *FLT3*-internal tandem duplication allelic ratio by next generation sequencing and fragment analysis

In comparing the allelic ratio obtained by next generation sequencing to the gold standard allelic ratio obtained by fragment analysis, 128/136 (94%) cases showed concordant allelic ratio classification following the 0.5 cutoff convention endorsed by the European Leukemia Net and National Comprehensive Cancer Network consensus guidelines. Fifty-three and seventy-five cases were classified as high and low allelic ratio, respectively. Of the eight discordant cases, all exhibited a high allelic ratio by fragment analysis ranging from 0.5 to 1.2, whereas the corresponding allelic ratio by next generation sequencing fell in the low category with 6 and 2 showing lower values of 0.4 and 0.3, respectively (Table 1). Their *FLT3*-internal tandem duplication sizes ranged from 18 to 87 bp, showing no predilection for large internal tandem duplication >100 bp. Their insertion sites involved exon 14c.1750–c.1827 where a major cluster of internal tandem duplications were observed (67% of all internal tandem duplications). This region was not associated with a high GC content (30%) nor long stretches of homopolymers (only one 4-mer present).

**Table 1:** *FLT3*-internal tandem duplication-positive cases with discordant allelic ratio classification by next generation sequencing and fragment analysis

Allelic ratio by fragment analysis	Allelic ratio by next generation sequencing	*FLT3*-internal tandem duplication detected by next generation sequencing	Internal tandem duplication size (bp)
1.2	0.4	c.1785_1837+1dup; p.?	54
0.7	0.4	c.1784_1804dup; p.R595_L601dup	21
0.5	0.4	c.1817_1818ins87; p.P606_R607ins29	87
0.5	0.4	c.1770_1793dup; p.Y597_E598ins8	24
0.8	0.4	c.1794_1795ins63; p.E598_Y599ins21	63
0.5	0.4	c.1750_1809dup; p.S584_W603dup	60
0.9	0.3	c.1764_1765ins18; p.E588_Y589insDPYIDP	18
0.5	0.3	c.1827_1828ins81; p.Gly583_Asn609dup	81

**Fig. 2:**
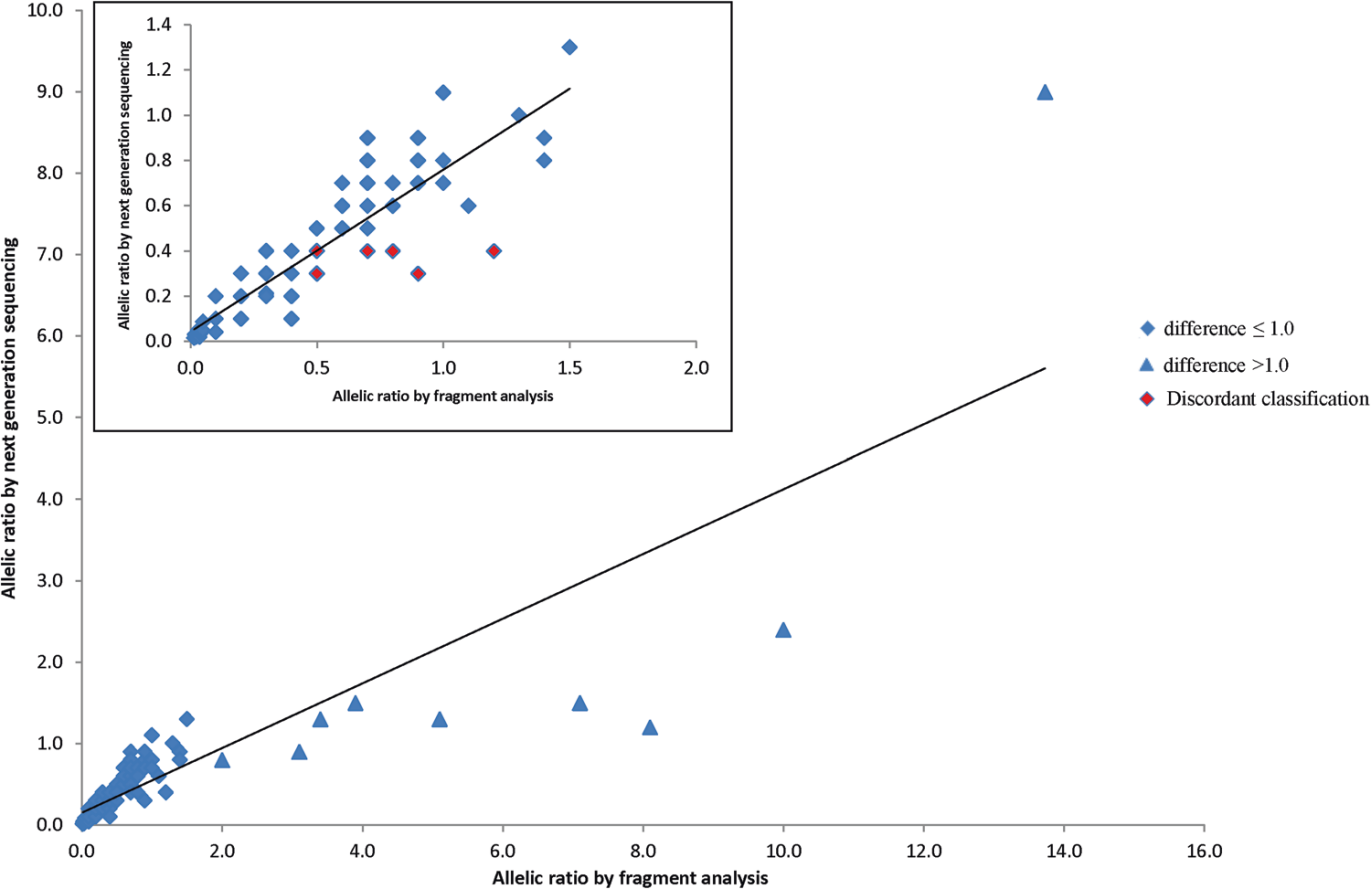
Comparison of *FLT3*-internal tandem duplication allelic ratio by next generation sequencing and fragment analysis in 136 dual-tested *FLT3*-internal tandem duplication-positive cases. Insert, zoomed-in comparison data of cases with fragment analysis allelic ratio <2.0

Overall, the allelic ratio by next generation sequencing positively correlated with the standard allelic ratio by fragment analysis with a Pearson correlation coefficient of 0.85 (*p* < 0.00001, 95% CI = 0.79–0.89, Fig. 2). By absolute values, the former was equal to, lower than, or higher than the fragment analysis allelic ratio in 51 (38%), 71 (52%), and 14 (10%) cases, respectively. There was no clear association between the degree of allelic ratio difference and size of *FLT3-*internal tandem duplication (Pearson correlation coefficient −0.15, *p* = 0.09, 95% CI = −0.31–0.02). Minimal absolute allelic ratio differences (≤0.1) were observed in 98/136 (72%) cases, with *FLT3*-internal tandem duplications spanning 3 –231 bp, and the allelic ratios by next generation and fragment analysis ranged from 0.02–1.1 and 0.02 to 1.0, respectively (Supplementary Table 1). Nine cases showed allelic ratio differences >1.0 (range 1.2–7.6), however all were concordantly classified as high allelic ratio, with the next generation sequencing and fragment analysis allelic ratios ranged from 0.8–9.0 to 2.0–13.7, respectively (Fig. 2, blue triangles). In all nine cases, the allelic ratio values were lower by next generation sequencing. Their *FLT3*-internal tandem duplication sizes were 21 (*n* = 2), 24 (*n* = 2), 39, 48, 69, 75, and 96 bp, exhibiting no correlation of large allelic ratio difference with large *FLT3*-internal tandem duplication >100 bp.

### Comparison of *FLT3*-tyrosine kinase domain mutation detection by next generation sequencing and fragment analysis

**Fig. 3:**
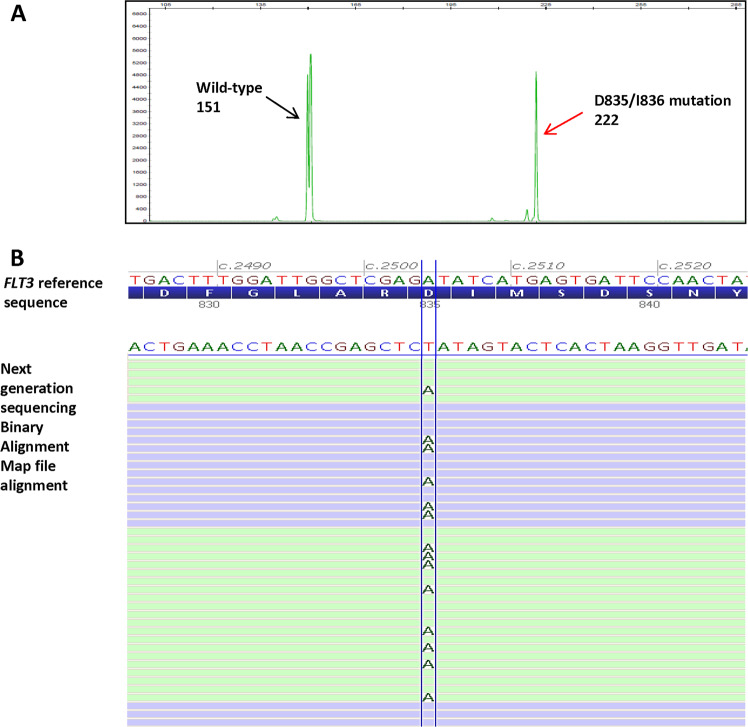
A representative acute myeloid leukemia case harboring an *FLT3*-tyrosine kinase domain mutation at D835/I836 tested by fragment analysis and next generation sequencing. **a** *FLT3* mutational analysis by fragment analysis test showing a tyrosine kinase domain mutation at D835/I836; *x* axis represent the PCR product size in base pair (bp) and *y* axis represent the fluorescence intensity. The wild-type peak at 151 bp (black arrow) successfully cleaved by restriction enzyme EcoRV and the mutant peak at 222 bp (red arrow) resistant to EcoRV cleavage were indicated. **b** Next generation sequencing read alignments displayed in Alamut® Visual revealed sequence of the corresponding missense mutation at D835/I836, c.2504 A>T; p.D835V. Note that the *FLT3* coding reference sequence is in reverse complementary orientation to the next generation sequencing Binary Alignment Map alignment sequence

*FLT3*-tyrosine kinase domain mutations were interrogated by both the next generation sequencing and fragment analysis methods. The next generation sequencing assay evaluates exons 15–20; whereas fragment analysis specifically assesses the mutational hotspot at codons D835/I836 in exon 20 using resistance to EcoRV digestion as a surrogate marker for a D835/I836 mutation (Fig. 3). Nineteen of the 402 dual-tested cases were positive for D835/I836 mutations by fragment analysis, including 16 substitutions, one 3 bp deletion and two 6 bp deletions. Eight of them also co-harbored an *FLT3*-internal tandem duplication (Table 2). In next generation sequencing, all 19 cases showed concordant results at D835/I836, with the 16 substitutions being D835Y (*n* = 11), D835V (*n* = 3), D835H (*n* = 2), D835N (*n* = 1) and 1 co-harboring D835N and D835V. Next generation sequencing confirmed the 3 and 6 bp deletions to be p.I836del and p.R834_D835del, respectively. All cases negative for D835/I836 mutations by fragment analysis were negative by next generation sequencing.

**Table 2:** Next generation sequencing findings of *FLT3*-tyrosine kinase domain mutations in cases positive for D835/I836 mutations by fragment analysis

Fragment analysis results	Next generation sequencing results
Positive D835/I836 substitution	c.2503 G>T; p.D835Y
Positive D835/I836 substitution	c.2503 G>T; p.D835Y
Positive D835/I836 substitution	c.2503 G>T; p.D835Y
Positive D835/I836 substitution	c.2503 G>T; p.D835Y
Positive D835/I836 substitution	c.2503 G>T; p.D835Y
Positive D835/I836 substitution	c.2504 A>T; p.D835V
Positive D835/I836 substitution	c.2503 G>C; p.D835H
Positive D835/I836 substitution	c.2503 G>T; p.D835Y
Positive D835/I836 substitution	c.2503 G>A; p.D835N;
	c.2504 A>T; p.D835V;
	c.2028 C>T; p.N676K
Positive D835/I836 substitution	c.2503 G>T; p.D835Y
Positive D835/I836 substitution	c.2503 G>T; p.D835Y
Positive D835/I836 substitution	c.2503 G>C; p.D835H;
and internal tandem duplication	c.1827_1828ins90; p.L610_E611ins30
Positive D835/I836 substitution	c.2503 G>T; p.D835Y;
and internal tandem duplication	c.1759_1788dup; p.N587_E596dup
Positive D835/I836 3 bp deletion	c.2508_2510del; p.I836del;
and internal tandem duplication	c.1754_1795dup; p.S585_E598dup
Positive D835/I836 6 bp deletion	c.2500_2505del; p.R834_D835del;
and internal tandem duplication	c.1744_1830dup; p.T582_L610dup
Positive D835/I836 6 bp deletion	c.2500_2505del; p.R834_D835del;
and internal tandem duplication	c.1744_1830dup; p.T582_L610dup
Positive D835/I836 substitution	c.2503 G>T; p.D835Y;
and internal tandem duplication	c.1786_1806dup; p.E596_K602dup
Positive D835/I836 substitution	c.2503 G>T; p.D835Y;
and internal tandem duplication	c.1743_1802dup; p.T582_L601dup
Positive D835/I836 substitution	c.2504 A>T; p.D835V;
and internal tandem duplication	c.1837_1837 + 1ins57; p.F612_G613ins19

### Additional *FLT3* mutations identified by next generation sequencing

Next generation sequencing detected seven additional mutations in regions not covered by the fragment analysis test (Table 3), including four juxtamembrane domain missense mutations p.V592A, p.V579A, p.V579I, and p.L576P, one juxtamembrane domain deletion p.Q577_Q580del, and two tyrosine kinase domain missense mutations p.D839A and p.N676K. V579 and V592 variants have been shown to be gain-of-function activing mutations with sensitivity to *FLT3* inhibitor midostaurin [27]. Although the function of L576P and Q577_Q580del has not been characterized, similar juxtamembrane domain mutations involving the same or neighboring amino acids were shown to be activating with sensitivity to *FLT3* inhibitor crenolanib and midostaurin/quizartinib, respectively [28]. The tyrosine kinase domain mutations N676K and D839A occur in the N-lobe and activation loop of *FLT3*, respectively. The former is activating with reported sensitivity to midostaurin, quizartinib, and crenolanib, however the sensitivity to the first two inhibitors was lost in the presence of *FLT3*-internal tandem duplication in in vitro assays [29, 30]. Acquisition of the activation loop mutation D839A in *FLT3*-internal tandem duplication mutant has also been shown to confer resistance to type II *FLT3* inhibitor PLX3397 [31]. All seven variants were therefore classified as pathogenic/likely pathogenic [32].

**Table 3:** Additional *FLT3* mutations identified by next generation sequencing in the 402 dual-tested cases

Fragment analysis results	Next generation sequencing results	Next generation sequencing variant allele fraction
Negative	c.1775T >C; p.V592A	18%
Negative	c.1736T >C; p.V579A	23%
Negative	c.1735G >A; p.V579I	43%
Negative	c.1727T >C; p.L576P	11%
Negative	c.1728_1739del; p.Q577_Q580del	5%
Negative	c.2516 A>C; p.D839A	9%
Positive for D835/I836 mutations	c.2503 G>A; p.D835N	24%
	c.2504 A>T, p.D835V	5%
	c.2028 C>A; p.N676K	11%

## Discussion

In this study, we systemically evaluated the performance of a custom-designed, hybridization capture-based, targeted next generation sequencing test in *FLT3* mutational analysis. To our knowledge, this is the largest comparative study to date of next generation sequencing and reference fragment analysis methods for *FLT3* mutation analysis. Such evaluation is critically required given the growing application of next generation sequencing testing as a comprehensive approach for myeloid malignancies, including acute myeloid leukemia. Our data confirmed the known heterogeneity of *FLT3*-internal tandem duplication, evidenced by its wide spectrum of sizes ranging from 3 to 231 bp and insertion sites involving exon 14, 15, and intron 14. Most importantly, next generation sequencing showed 100% concordance with the fragment analysis assay in *FLT3*-internal tandem duplication detection, illustrating that *FLT3*-internal tandem duplication can be reliably detected by next generation sequencing using hybridization capture-based chemistry methods, optimized bioinformatics, and dedicated analysis. Detection of *FLT3*-internal tandem duplication by next generation sequencing is known to be challenging, mainly because the standard bioinformatics algorithms are not optimized for larger insertion/deletion (>20 bp) detection. Amplicon-based next generation sequencing faces additional design challenges that compromise adequate sequence coverage of the *FLT3-*internal tandem duplication/wild-type junctions in large *FLT3*-internal tandem duplications, and the uniform amplicon start-stop positions can further complicate bioinformatics algorithms for indel detection. These factors account for the variable success of *FLT3*-internal tandem duplication detection that has been reported [33–38]: it has been detected by hybridization capture-based next generation sequencing at 102–185 bp and less impressively by amplicon-based chemistry at 60–126 bp. The 231 bp *FLT3*-internal tandem duplication identified in our study is larger than previous reports, further supporting the superiority of hybridization capture-based chemistry in *FLT3*-internal tandem duplication detection by next generation sequencing. The lack of larger *FLT3*-internal tandem duplications in our study cohort is likely attributable to the extreme rarity of *FLT3*-internal tandem duplication >200 bp [39]; however we are confident these events can be accurately detected as evidenced by our recent successful identification of a 416 bp duplication in the *STAG2* gene (data not shown) using our next generation sequencing panel.

In our next generation sequencing assay, as the default aligner and variant caller CLC Bio was only able to accurately detect and call *FLT3*-internal tandem duplications up to 30 bp, we supplemented the *FLT3*-internal tandem duplication assessment with the in-house breakpointSearch tool and manual review. The *FLT3*-internal tandem duplication regions (exons 14–15) were manually reviewed in all cases to confirm the presence/absence of *FLT3*-internal tandem duplication reported by the breakpointSearch tool and the *FLT3*-internal tandem duplication variant calls made by CLC-Bio (internal tandem duplication <30 bp), and to make the final *FLT3*-internal tandem duplication variant call in internal tandem duplications ≥30 bp. As the vast majority of *FLT3*-internal tandem duplications present as complete or near complete duplications (98% in our study cohort), the manual review process is relatively straightforward: *FLT3*-internal tandem duplications <30 bp were correctly aligned and called by CLC Bio and readily confirmed in the Binary Alignment Map files; for *FLT3*-internal tandem duplications ≥30 bp, the duplicated wild-type sequences irrespective of sizes were flanked by the 5′ and 3′ soft-clipped bases with the latter marking the *FLT3*-internal tandem duplication insertion site and revealing the *FLT3*-internal tandem duplication sequences (Fig. 1b). This study convincingly demonstrated that our bioinformatics platform supplemented with the breakpointSearch tool reliably detects *FLT3*-internal tandem duplication. This excellent bioinformatic accuracy suggests that it may be a viable option to reserve the manual *FLT3*-internal tandem duplication review process for the small percentage of cases flagged for its presence by the breakpointSearch tool (335/7902, 4% in our study) and thereby reduce case analysis time; however, our practice is to utilize the algorithmic solution in conjunction with manual review in all cases.

The allelic ratio of *FLT3*-internal tandem duplication obtained by next generation sequencing positively correlated with that obtained by fragment analysis and the majority showed minimal absolute differences ≤0.1. Importantly, 94% cases showed concordant allelic ratio classification used in acute myeloid leukemia risk stratification and the remaining demonstrated borderline low allelic ratio values of 0.3 and 0.4 by next generation sequencing. These findings illustrate that allelic ratio obtained by our next generation sequencing assay reliably classifies the vast majority of *FLT3*-internal tandem duplications, which is an important consideration in light of current guidelines for the prognosis and management of *FLT3-*internal tandem duplication-positive acute myeloid leukemia [10, 11]. However, for a borderline next generation sequencing allelic ratio of 0.3–0.4, it would be prudent to confirm using fragment analysis if clinically indicated in the context of other clinical and laboratory findings.

One interesting finding from the allelic ratio comparison was that relatively large differences >1.0 were observed in cases with high allelic ratio ≥2.0 by fragment analysis, with all nine cases showing lower allelic ratios by next generation sequencing although all were concordantly classified in the high category by both methods (Fig. 2). The large difference seen in these high allelic ratio cases was likely attributable to the disparity of the testing platforms and how allelic ratio was assessed in each assay. In fragment analysis, it was calculated from the fluorescent intensity ratio of the *FLT3*-internal tandem duplication versus wild-type peaks obtained from capillary electrophoresis (Fig. 1), whereas in next generation sequencing it was calculated from the ratio of the number of sequencing reads showing *FLT3*-internal tandem duplication versus wild-type sequences. As the next generation sequencing read alignment and display are subject to bioinformatics pipeline quality processing and filtering, potentially informative reads flagged with sequence quality below set thresholds may be discarded, leading to a bias toward a lower *FLT3*-internal tandem duplication/wild-type allelic ratio by next generation sequencing. This bias becomes more exaggerated in cases with higher allele burden (and higher allelic ratio) (Supplementary Table 2): e.g., a 10% variant allele fraction difference between 10 and 20% would result in an allelic ratio difference of 0.2, whereas the same 10% variant allele fraction change from 70 to 80% or 80 to 90% would lead to a significant allelic ratio difference of 1.7 or 5.0, respectively. Although large allelic ratio differences were seen in high allelic ratio cases in our study cohort, it is worth noting that these differences had no impact on the high and low classification of allelic ratio and therefore did not impact acute myeloid leukemia risk stratification clinically. Furthermore, all nine cases with >1.0 differences had *FLT3*-internal tandem duplication sizes <100 bp, showing no bias of greater allelic ratio difference in large *FLT3*-internal tandem duplication >100 bp.

Next generation sequencing also provided more comprehensive information than fragment analysis. Although the most common juxtamembrane domain mutation is *FLT3*-internal tandem duplication, missense mutations or small deletions in this region have been shown to be activating with demonstrated responsiveness to FLT3 inhibitors, suggesting that patients harboring these mutations may benefit from FLT3 inhibition [27, 28]. Five such cases detected by next generation sequencing were missed by fragment analysis as the latter only assesses the presence/absence of *FLT3*-internal tandem duplication in the juxtamembrane region (Table 3). Similarly, two non-D835/I836 tyrosine kinase domain mutations were identified by next generation sequencing only and their detection may guide the choice of FLT3 inhibitors in the context of *FLT3*-internal tandem duplication. Furthermore, the specific variant sequences revealed by next generation sequencing provide basis for future high-sensitivity, sequence-specific, minimal residual disease monitoring and may guide FLT3 inhibitor choice in clinical trials as D835 mutations may confer differential resistance to type II FLT3 inhibitors [40].

In summary, we present a large study comparing clinical *FLT3* testing by next generation sequencing and fragment analysis. Using a hybridization capture-based chemistry and optimized bioinformatics pipeline, we show that next generation sequencing can reliably detect *FLT3*-internal tandem duplication and classify its allelic ratio for acute myeloid leukemia risk stratification. Next generation sequencing exhibited superior comprehensiveness in *FLT3* genetic alteration assessment, providing additional information in regions not examined by fragment analysis, which may further improve personalized, targeted therapy in acute myeloid leukemia. Finally, as next generation sequencing panels are commonly used at acute myeloid leukemia diagnosis to evaluate multiple clinically relevant markers, next generation sequencing may be the sole *FLT3* test required if its performance can demonstrate comparability to the standard fragment analysis test, and the turn-around-time can meet clinical needs. Otherwise, fragment analysis should be performed along with next generation sequencing to ensure prompt initiation of FLT3 inhibitor therapy in acute myeloid leukemia.

## Supplementary information

Supplementary material
