# Correction to: Hybridization capture-based next-generation sequencing reliably detects *FLT3* mutations and classifies *FLT3*-internal tandem duplication allelic ratio in acute myeloid leukemia: a comparative study to standard fragment analysis

**DOI:** 10.1038/s41379-019-0378-6

**Published:** 2019-10-07

**Authors:** Rong He, Daniel J. Devine, Zheng Jin Tu, Ming Mai, Dong Chen, Phuong L. Nguyen, Jennifer L. Oliveira, James D. Hoyer, Kaaren K. Reichard, Paul L. Ollila, Aref Al-Kali, Ayalew Tefferi, Kebede H. Begna, Mrinal M. Patnaik, Hassan Alkhateeb, David S. Viswanatha

**Affiliations:** 1 Division of Hematopathology, Mayo Clinic College of Medicine, Rochester, MN USA; 2 Biomedical statistics and informatics, Mayo Clinic College of Medicine, Rochester, MN USA; 3 Division of Hematology, Mayo Clinic College of Medicine, Rochester, MN USA; 4 Present Address: Department of Laboratory Medicine, Cleveland Clinic, Cleveland, OH USA

**Keywords:** Acute myeloid leukaemia, Prognostic markers

**Correction to: Modern Pathology**

10.1038/s41379-019-0359-9

This article was erroneously published with standard licence and copyright, but it is in fact published with open access, so has a CC BY licence and the copyright line is ‘The Author(s)’. This has been updated in the original article.

