# Supplementary material for: Hybridization capture-based next generation sequencing reliably detects FLT3 mutations and classifies FLT3-internal tandem duplication allelic ratio in acute myeloid leukemia: a comparative study to standard fragment analysis
Source: Mod Pathol. 2019 Aug 30;33(3):334–43. doi: 10.1038/s41379-019-0359-9 (PMC7051912; doi:10.1038/s41379-019-0359-9)

Supplementary Table 1. Allelic ratio of *FLT3*-internal tandem duplication by fragment analysis and next generation sequencing in the 136 dual-tested positive cases.

| Allelic ratio by fragment analysis | Allelic ratio by next generation sequencing | Allelic ratio concordance | Allelic ratio differences | *FLT3*-internal tandem duplication Identified by next generation sequencing | *FLT3*-internal tandem duplication Size (bp) |
| --- | --- | --- | --- | --- | --- |
| 0.3 | 0.2 | Y | 0.1 | c.1784_1804dup, p.Arg595_Leu601dup | 21 |
| 0.2 | 0.1 | Y | 0.1 | c.1786_1806dup, p.Glu596_Lys602dup | 21 |
| 0.7 | 0.5 | Y | 0.2 | c.1770_1835dup, p.Tyr591_Phe612dup | 66 |
| 0.8 | 0.6 | Y | 0.2 | c.1777_1800dup, p.Asp593_Asp600dup | 24 |
| 0.6 | 0.5 | Y | 0.1 | c.1827_1828ins66, p.Asn609_Leu610ins22 | 66 |
| 0.4 | 0.1 | Y | 0.3 | c.1827_1828ins60, p.Asn609_Leu610ins20 | 60 |
| 5.1 | 1.3 | Y | 3.8 | c.1758_1832dup, p.Asp586_Leu610dup | 75 |
| 0.1 | 0.1 | Y | 0.0 | c.1780_1824dup, p.Phe594_Glu608dup | 45 |
| 0.5 | 0.5 | Y | 0.1 | c.1770_1793dup, p.Tyr597_Glu598ins8 | 24 |
| 3.4 | 1.3 | Y | 2.1 | c.1742_1810dup, p.Val581_Trp603dup | 69 |
| 0.7 | 0.5 | Y | 0.2 | c.1759_1785dup, p.Asn587_Arg595dup | 27 |
| 0.7 | 0.9 | Y | -0.2 | c.1780_1797dup, p.Phe594_Tyr599dup | 18 |
| 0.9 | 0.8 | Y | 0.2 | c.1779_1793dup, p.Asp593_Tyr597dup | 15 |
| 0.2 | 0.2 | Y | 0.0 | c.1778_1891dup, p.Tyr630_Gly631ins68 | 204 |
| 0.7 | 0.5 | Y | 0.2 | c.1837_1837+1ins66, p.Phe612_Gly613ins22 | 66 |
| 0.4 | 0.4 | Y | 0.0 | c.1777_1803dup, p.Asp593_Leu601dup | 27 |
| 0.3 | 0.3 | Y | 0.0 | c.1793_1794ins63, p.Glu598_Tyr599ins21 | 63 |
| 0.1 | 0.1 | Y | 0.0 | c.1796_1852dup, p.Gly617_Ser618ins49 | 147 |
| 1.2 | 0.4 | N | 0.8 | c.1785_1837+1dup, p.? | 54 |
| 0.05 | 0.05 | Y | 0.0 | c.1736_1804dup, p.Leu601_Lys602ins23 | 69 |
| 8.1 | 1.2 | Y | 6.9 | c.1770_1793dup, p.Tyr597_Glu598ins8 | 24 |
| 0.1 | 0.1 | Y | 0.0 | c.1779_1832dup, p.Asp593_Leu610dup | 54 |
| 0.4 | 0.2 | Y | 0.2 | c.1780_1800dup, p.Phe594_Asp600dup | 21 |
| 0.5 | 0.4 | Y | 0.2 | c.1783_1784ins42, p.Phe594_Arg595ins14 | 42 |
| 0.1 | 0.1 | Y | 0.0 | c.1770_1835dup, p.Tyr591_Phe612dup | 66 |
| 0.05 | 0.1 | Y | -0.05 | c.1738_1812dup, p.Gln580_Glu604dup | 75 |
| 0.1 | 0.1 | Y | 0.0 | c.1820_1821ins54, p.Pro606_Arg607ins18 | 54 |
| 0.2 | 0.2 | Y | 0.0 | c.1795_1796ins78, p.Glu598_Tyr599ins26 | 78 |
| 0.4 | 0.3 | Y | 0.1 | c.1795_1818dup, p.Tyr599_Pro606dup | 24 |
| 0.6 | 0.6 | Y | 0.0 | c.1731_1793dup, p.Met578_Glu598dup | 63 |
| 1.5 | 1.3 | Y | 0.2 | c.1742_1831dup, p.Val581_Leu610dup | 90 |
| 0.1 | 0.1 | Y | 0.0 | c.1731_1793dup, p.Met578_Glu598dup | 63 |
| 0.7 | 0.4 | N | 0.3 | c.1784_1804dup, p.Arg595_Leu601dup | 21 |
| 0.1 | 0.1 | Y | 0.0 | c.1866_1867ins231, p.Gly622_Lys623ins77 | 231 |
| 0.04 | 0.04 | Y | 0.0 | c.1782_1783ins33, p.Ser584_Phe594dup | 33 |
| 0.1 | 0.1 | Y | 0.1 | c.1778_1779ins27, p.Val592_Asp593ins9 | 27 |
| 0.5 | 0.4 | N | 0.1 | c.1817_1818ins87, p.Pro606_Arg607ins29 | 88 |
| 0.01 | 0.02 | Y | 0.0 | c.1790_1813dup, p.Tyr597_Glu604dup | 24 |
| 0.5 | 0.4 | N | 0.1 | c.1770_1793dup, p.Tyr597_Glu598ins8 | 24 |
| 0.1 | 0.1 | Y | 0.0 | c.1759_1800dup, p.Asn587_Asp600dup | 42 |
| 0.1 | 0.1 | Y | 0.0 | c.1794_1795ins63, p.Glu598_Tyr599ins21 | 63 |
| 0.1 | 0.2 | Y | -0.1 | c.1730_1801dup, p.Gln577_Asp600dup | 72 |
| 0.1 | 0.1 | Y | 0.0 | c.1833_1834ins96, p.Glu611_Phe612ins32 | 96 |
| 0.7 | 0.7 | Y | 0.0 | c.1832_1833ins117, p.Leu610_Glu611ins39 | 117 |
| 0.7 | 0.8 | Y | -0.1 | c.1785_1837+1dup, p.? | 54 |
| 0.5 | 0.5 | Y | 0.0 | c.1871_1872ins216, p.Val624_Met625ins72 | 216 |
| 0.2 | 0.3 | Y | -0.1 | c.1785_1835dup, p.Glu611_Phe612ins17 | 51 |
| 0.8 | 0.4 | N | 0.4 | c.1794_1795ins63, p.Glu598_Tyr599ins21 | 63 |
| 0.6 | 0.6 | Y | 0.0 | c.1861_1862ins183, p.Ala620_Phe621ins61 | 183 |
| 0.5 | 0.5 | Y | 0.0 | c.1861_1862ins183, p.Ala620_Phe621ins61 | 183 |
| 1.4 | 0.9 | Y | 0.5 | c.1786_1787ins42, p.Arg595_Glu596ins14 | 42 |
| 0.5 | 0.4 | N | 0.1 | c.1750_1809dup, p.Ser584_Trp603dup | 60 |
| 0.04 | 0.02 | Y | 0.02 | c.1837+12_1837+13ins84, p.? | 84 |
| 3.1 | 0.9 | Y | 2.1 | c.1748_1786dup, p.Gly583_Arg595dup | 39 |
| 0.02 | 0.02 | Y | 0.0 | c.1830_1831ins99, p.Leu610_Glu611ins33 | 99 |
| 0.2 | 0.2 | Y | 0.0 | c.1837+3_1837+4ins99, p.? | 99 |
| 0.9 | 0.3 | N | 0.6 | c.1764_1765ins18, p.Glu588_Tyr589insAspProTyrIleAspPro | 18 |
| 7.1 | 1.5 | Y | 5.6 | c.1770_1793dup, p.Tyr597_Glu598ins8 | 24 |
| 0.5 | 0.5 | Y | 0.0 | c.1737_1778dup, p.Val592_Asp593ins14 | 42 |
| 0.04 | 0.04 | Y | 0.0 | c.1837+15_1837+16ins87, p.? | 87 |
| 0.5 | 0.3 | Y | 0.2 | c.1783_1830dup, p.Arg595_Leu610dup | 48 |
| 0.4 | 0.4 | Y | 0.0 | c.1833_1834ins63, p.Glu611_Phe612ins21 | 63 |
| 1.4 | 0.8 | Y | 0.6 | c.1720_1788dup, p.Ser574_Glu596dup | 69 |
| 0.2 | 0.2 | Y | 0.0 | c.1792_1837+2dup, p.? | 48 |
| 0.2 | 0.2 | Y | 0.0 | c.1816_1817ins42, p.Phe605_Pro606ins14 | 42 |
| 0.2 | 0.2 | Y | 0.0 | c.1820_1821ins54, p.Pro606_Arg607ins18 | 54 |
| 0.5 | 0.5 | N | 0.1 | c.1770_1787dup, p.Arg595_Glu596insAspTyrValAspPheArg | 18 |
| 0.02 | 0.03 | Y | -0.01 | c.1822_1823ins93, p.Arg607_Glu608ins31 | 93 |
| 0.1 | 0.1 | Y | 0.0 | c.1773_1805dup, p.Leu601_Lys602ins11 | 33 |
| 1.1 | 0.6 | Y | 0.5 | c.1807_1836dup, p.Trp603_Phe612dup | 30 |
| 0.2 | 0.3 | Y | -0.1 | c.1797_1798ins18, p.Tyr599_Asp600insSerProProPheProTyr | 18 |
| 0.6 | 0.6 | Y | 0.0 | c.1736_1810dup, p.Val579_Trp603dup | 75 |
| 0.04 | 0.04 | Y | 0.0 | c.1731_1787dup, p.Met578_Glu596dup | 57 |
| 0.3 | 0.4 | Y | -0.1 | c.1729_1794dup, p.Gln577_Glu598dup | 66 |
| 0.02 | 0.02 | Y | 0.0 | c.1774_1794dup, p.Val592_Glu598dup | 21 |
| 13.7 | 9.0 | Y | 4.7 | c.1789_1809dup, p.Tyr597_Trp603dup | 21 |
| 0.7 | 0.6 | Y | 0.1 | c.1751_1786dup, p.Arg595_Glu596ins12 | 36 |
| 0.7 | 0.5 | Y | 0.2 | c.1784_1804dup, p.Arg595_Leu601dup | 21 |
| 0.3 | 0.2 | Y | 0.1 | c.1784_1785ins30, p.Asp586_Arg595dup | 30 |
| 0.2 | 0.1 | Y | 0.1 | c.1756_1794dup, p.Asp586_Glu598dup | 39 |
| 0.7 | 0.5 | Y | 0.2 | c.1779_1793dup, p.Asp593_Tyr597dup | 15 |
| 0.8 | 0.6 | Y | 0.2 | c.1780_1800dup, p.Phe594_Asp600dup | 21 |
| 3.9 | 1.5 | Y | 2.4 | c.1792_1837+2dup, p.? | 48 |
| 0.2 | 0.1 | Y | 0.1 | c.1837+2_1837+3ins48, p.? | 48 |
| 0.7 | 0.5 | Y | 0.2 | c.1820_1821ins48, p.Arg607_Glu608ins16 | 48 |
| 0.6 | 0.5 | Y | 0.1 | c.1798_1833dup, p.Asp600_Glu611dup | 36 |
| 0.03 | 0.03 | Y | 0.0 | c.1824_1825ins60, p.Glu608_Asn609ins20 | 60 |
| 0.7 | 0.5 | Y | 0.2 | c.1795_1796ins27, p.Glu598_Tyr599ins9 | 27 |
| 0.03 | 0.03 | Y | 0.0 | c.1841_1842ins138, p.Gly613_Lys614ins46 | 138 |
| 0.8 | 0.6 | Y | 0.2 | c.1816_1817ins42, p.Phe605_Pro606ins14 | 42 |
| 0.4 | 0.4 | Y | 0.0 | c.1827_1828ins81; p.Gly583_Asn609dup, | 42 |
| 0.6 | 0.5 | Y | 0.1 | c.1833_1834ins51; p.Glu611_Phe612ins17 , | 51 |
| 2.0 | 0.8 | Y | 1.2 | c.1837+4_1837+5ins96, p.? | 96 |
| 0.7 | 0.6 | Y | 0.1 | c.1770_1787dup, p.Arg595_Glu596insAspTyrValAspPheArg | 18 |
| 1.3 | 1.0 | Y | 0.3 | c.1784_1825dup, p.Arg595_Glu608dup | 41 |
| 0.2 | 0.1 | Y | 0.1 | c.1759_1788dup, p.Asn587_Glu596dup | 30 |
| 0.5 | 0.3 | N | 0.2 | c.1827_1828ins81, p.Gly583_Asn609dup | 81 |
| 0.9 | 0.9 | Y | 0.0 | c.1779_1805dup, p.Leu601_Lys602ins9 | 27 |
| 0.4 | 0.4 | Y | 0.0 | c.1827_1828ins90, p.Leu610_Glu611ins30 | 90 |
| 0.2 | 0.2 | Y | 0.0 | c.1806_1807ins24, p.Lys602_Trp603ins8 | 24 |
| 0.3 | 0.3 | Y | 0.0 | c.1796_1837dup, p.Phe612_Gly613ins14 | 42 |
| 0.1 | 0.04 | Y | 0.06 | c.1789_1809dup, p.Tyr597_Trp603dup | 21 |
| 0.7 | 0.6 | Y | 0.1 | c.1773_1799dup, p.Val592_Asp600dup | 27 |
| 0.6 | 0.6 | Y | 0.0 | c.1827_1828ins111; p.Asn609_Leu610ins37 | 111 |
| 0.3 | 0.2 |  | 0.1 | C.1785_1811dup; p.Glu596_Glu604dup | 27 |
| 0.8 | 0.6 | Y | 0.2 | c.1770_1793dup, p.Tyr597_Glu598ins8 | 33 |
| 0.02 | 0.03 | Y | -0.01 | c.1778_1813dup, p.Glu604_Phe605ins12 | 36 |
| 0.8 | 0.7 | Y | 0.1 | c.1817_1818ins33, p.Pro606_Arg607ins11 | 33 |
| 0.1 | 0.1 | Y | 0.0 | c.1783delins16, p.Phe594_Arg595insProAspValThrThr | 15 |
| 0.2 | 0.1 | Y | 0.1 | c.1801_1802ins63, p.Asp600_Leu601ins21 | 63 |
| 0.4 | 0.3 | N | -0.1 | c.1789_1875dup, p.Met625_Asn626ins59 | 177 |
| 0.1 | 0.1 | Y | 0.0 | c.1829_1830ins78, p.Asn609_Leu610ins26; c.1740_1837+1dup, p.? | 78;  99 |
| 0.4 | 0.4 | Y | 0.0 | c.1837+1_1837+2ins78, p.? | 78 |
| 10 | 2.4 | Y | 7.6 | c.1780_1800dup, p.Phe594_Asp600dup | 21 |
| 0.03 | 0.02 | Y | 0.01 | c.1759_1812dup, p.Asn587_Glu604dup | 54 |
| 0.04 | 0.04 | Y | 0.0 | c.1783delins16, p.Phe594_Arg595insProAspValThrThr | 15 |
| 0.8 | 0.7 | Y | 0.1 | c.1770_1793dup, p.Tyr597_Glu598ins8 | 24 |
| 0.3 | 0.3 | Y | 0.0 | c.1794_1795ins15, p.Glu598_Tyr599insGlySerGluTyrGlu | 15 |
| 0.4 | 0.3 | Y | 0.1 | c.1784_1804dup, p.Arg595_Leu601dup;  c.1767_1811dup, p.Trp603_Glu604ins15 | 21;  45 |
| 0.04 | 0.03 | Y | 0.01 | c.1776_1802dup, p.Asp593_Leu601dup | 27 |
| 1.0 | 0.8 | Y | 0.2 | c.1763_1837dup, p.Glu588_Phe612dup | 75 |
| 0.03 | 0.02 | Y | 0.01 | c.1784_1804dup, p.Arg595_Leu601dup | 21 |
| 0.7 | 0.5 | Y | 0.2 | c.1828_1829ins60, p.Asn609_Leu610ins20 | 60 |
| 0.4 | 0.4 | Y | 0.0 | c.1831_1832ins87, p.Leu610_Glu611ins29; c.1753_1803dup, p.Ser585_Leu601dup; c.1823_1824ins51, p.Arg607_Glu608ins17; c.1795_1796ins66, p.Glu598_Tyr599ins22 | 87; 51; 51; 66 |
| 0.03 | 0.03 | Y | 0.0 | c.1789_1790ins21, p.Glu596_Tyr597insPheHisValAspPheArgGlu | 21 |
| 0.4 | 0.4 | Y | 0.0 | c.1792_1827dup, p.Glu598_Asn609dup | 36 |
| 1.0 | 0.7 | Y | 0.3 | c.1837+2_1837+3ins36, p.? | 36 |
| 0.2 | 0.3 | Y | -0.1 | c.1775_1888dup, p.Ala629_Tyr630ins68 | 204 |
| 0.9 | 0.7 | Y | 0.2 | c.1795_1815dup, p.Tyr599_Phe605dup | 21 |
| 0.4 | 0.3 | Y | 0.1 | c.1770_1793dup, p.Tyr597_Glu598ins8 | 24 |
| 0.6 | 0.7 | Y | -0.1 | c.1826_1827ins66; p.Asn609_Leu610ins22 | 66 |
| 0.7 | 0.6 | Y | 0.1 | c.1796_1816dup, p.Phe605_Pro606ins7; c.1745_1837+6dup, p.?; c.1738_1818dup, p.Gln580_Pro606dup | 21; 99;  81 |
| 0.05 | 0.1 | Y | -0.05 | c.1855_1856ins186;p.Ser618_Gly619ins62 | 186 |
| 0.2 | 0.1 | Y | 0.1 | c.1754_1795dup; p.Ser585_Glu598dup | 42 |
| 1.0 | 1.1 | Y | -0.1 | c.1849_1850ins168; p.Leu616_Gly617ins56 | 168 |
| 0.2 | 0.2 | Y | 0.0 | c.1785_1786insCGA, p.Arg595dup | 3 |

Supplementary Table 2. Conversion table of *FLT3*-internal tandem duplication variant allele fraction and allelic ratio.

| **Variant allele fraction** | **Allelic ratio** |
| --- | --- |
| 5% | 5/95 = 0.05 |
| 10% | 10/90 = 0.1 |
| 20% | 20/80 = 0.3 |
| 30% | 30/70 = 0.4 |
| 40% | 40/60 = 0.7 |
| 50% | 50/50 = 1.0 |
| 60% | 60/40 = 1.5 |
| 70% | 70/30 = 2.3 |
| 80% | 80/20 = 4.0 |
| 90% | 90/10 = 9.0 |
| 95% | 95/5 = 19.0 |

Supplementary Figure 1. Schematic diagram of the breakpointSearch tool work-flow for large insertion/deletion detection. Five main steps were developed to collect next generation sequencing reads with soft-clipped bases and then do *de novo* assembly to generate contigs. These contigs were then aligned to reference genome to identify source of indel located.


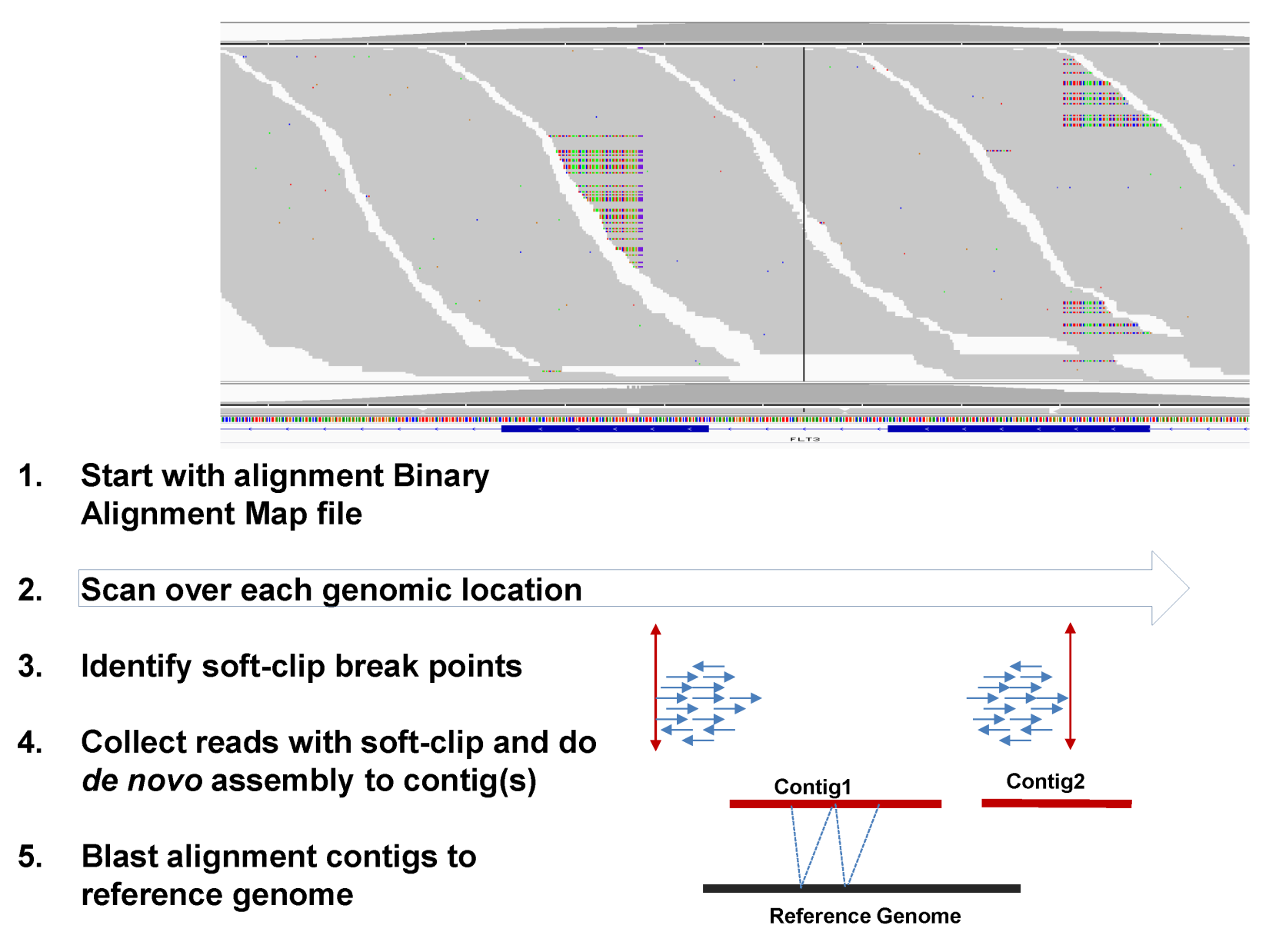

Supplement: Supplementary file 1 — Supplementary material [file 41379_2019_359_MOESM1_ESM.docx]
